# Landsat-Derived Estimates of Mangrove Extents in the Sierra Leone Coastal Landscape Complex during 1990–2016

**DOI:** 10.3390/s18010012

**Published:** 2017-12-21

**Authors:** Pinki Mondal, Sylwia Trzaska, Alex de Sherbinin

**Affiliations:** Center for International Earth Science Information Network (CIESIN), the Earth Institute at Columbia University, Palisades, NY 10964, USA; strzaska@ciesin.columbia.edu (S.T.); adesherbinin@ciesin.columbia.edu (A.d.S.)

**Keywords:** mangrove, spatiotemporal change, low elevation coastal zone, Landsat, West Africa

## Abstract

This study provides the first assessment of decadal changes in mangrove extents in Sierra Leone. While significant advances have been made in mangrove mapping using remote sensing, no study has documented long-term changes in mangrove extents in Sierra Leone—one of the most vulnerable countries in West Africa. Such understanding is critical for devising regional management strategies that can support local livelihoods. We utilize multi-date Landsat data and cloud computational techniques to quantify spatiotemporal changes in land cover, with focus on mangrove ecosystems, for 1990–2016 along the coast of Sierra Leone. We specifically focus on four estuaries—Scarcies, Sierra Leone, Yawri Bay, and Sherbro. We relied on the k-means approach for an unsupervised classification, and validated the classified map from 2016 using ground truth data collected from Sentinel-2 and high-resolution images and during field research (accuracy: 95%). Our findings indicate that the Scarcies river estuary witnessed the greatest mangrove loss since 1990 (45%), while the Sierra Leone river estuary experienced mangrove gain over the last 26 years (22%). Overall, the Sierra Leone coast lost 25% of its mangroves between 1990 and 2016, with the lowest coverage in 2000, during the period of civil war (1991–2002). However, natural mangrove dynamics, as supported by field observations, indicate the potential for regeneration and sustainability under carefully constructed management strategies.

## 1. Introduction

Mangroves are located in the tropical and sub-tropical countries primarily between 30° N and 30° S latitude. These coastal forests are distributed in the inter-tidal regions, and along river banks and lagoons. This ecosystem is comprised of plant families with specialized adaptations to live in the tidal environment. According to an estimate from 2000, mangrove forests accounted for less than 1% of total tropical forests in the world [1]. Yet, these are one of the most productive and biologically complex ecosystems that store three to four times more carbon per equivalent area compared to tropical forests [2]. Besides, mangrove forests provide protection to coastal communities from natural disasters, especially storm surge and small to moderate tsunamis [3,4]. However, due to increasing land competition for agriculture, aquaculture, tourism, and infrastructure development, these forests have declined from 18.8 million hectares in 1990 to 15.2 million hectares in 2005 [5]. 

Official estimates for the year 2000 suggest that Sierra Leone had 105,300 hectares of mangroves, or roughly 0.007% of the global total [5]. In this West African country, mangroves are an essential source of wood for the coastal communities and provide a number of indirect services such as fish breeding sites and coastal protection. Despite their undeniable benefits, mangrove forests are under increasing pressure due to urbanization and land reclamation on the flood plains, conversion to rice paddies and unsustainable exploitation for fuelwood and fish smoking. This fragile ecosystem is also sensitive to changing environmental conditions such as increased temperature and sea level rise, as well as water characteristics such as salinity, pollutants, and sedimentation [6]. The combination of increasing human-induced and environmental stress may lead to unsustainable conditions for mangroves and ultimately their decline. A better understanding of the recent changes in mangrove extent and quality, human pressures, the impact of climate change as well as management practices and opportunities may help sustaining mangroves and their benefits for future generations. However, up-to-date, good quality in situ data about mangroves are not available in Sierra Leone, hindering assessment of changes and the design of sustainable management plans. Remote sensing can be an alternative tool in this context, given the availability of free satellite data dating from the 1970s at spatial and temporal scales suitable for landscape-level monitoring. Taking advantage of these satellite images, this study aims at assessing landscape-level changes in mangrove extents in Sierra Leone during 1990–2016. 

Remote sensing tools and techniques have been widely used in mangrove mapping, and have rapidly evolved over the past decade [7,8,9]. One of the most widely used satellite sensors for mangrove mapping is Landsat, due to its spatial and temporal coverage, and ease of accessibility. Many studies have used Landsat images, as well as optical imagery from SPOT, MODIS, ASTER, QuickBird, WorldView, and IKONOS, for quantifying mangrove extents and spatiotemporal changes across the globe [1,10,11,12,13,14,15,16,17,18,19,20,21,22,23,24,25,26,27,28,29,30,31,32,33,34,35,36,37,38,39,40]. These studies use a range of classification techniques and machine-learning algorithms such as unsupervised, supervised, hybrid, classification and regression tree (CART), support vector machine (SVM), object-oriented classification among others. 

The currently available global mangrove estimates are either directly calculated from optical satellite data, national-level statistics, or are derived from other global datasets [1,5,41,42,43]. Most of these studies provide only a snapshot for mangrove extents because of the massive scale of work involved in global mapping efforts. Besides, it is difficult to directly compare estimates from these studies, ranging from 12 to 20 million hectares (see [5] for details) due to the differences in methods involved. Hamilton and Casey [41] identify that there are notable differences in mangrove estimates as provided by these global studies, and hence developed a new global dataset, CGMFC-21, that provides annual mangrove estimates for 2000–2012 by compiling other existing datasets [1,42,44] and using statistical techniques to predict estimates for 2013–2014. However, national/regional estimates should be derived from direct observation (either via satellite, or field visits), as these estimates are often used for revising national/regional policies with impacts on local livelihoods.

Many studies have reported spatiotemporal changes in mangroves from different parts of the world, especially the Sundarbans in India and Bangladesh, and West-Central Africa [12,13,14,25,26,31]. Past studies have reported widely variable single-date mangrove estimates for Sierra Leone, mostly owing to the different methodologies involved in these studies (Table 1). For the year 2000 alone, the estimates range from 655.67 km^2^ to 2917.01 km^2^ [41]. Even studies relying solely on Landsat images report a wide range of estimates [1,45], likely because different definitions of ‘mangrove forests’ were used in these studies. However, no study, to the best of our knowledge, has focused on long-term changes in Sierra Leone despite the importance of mangroves in providing coastal protection and livelihood opportunities in this vulnerable nation. Moreover, as one of the West African countries selected for the USAID-funded West Africa Biodiversity and Climate Change (WA BiCC) project, a detailed land cover change analysis over the past decades is now required for Sierra Leone in order to develop coastal conservation and climate resilience building activities. 

In this study, we provide the first multi-year assessment (1990, 2000, 2010, and 2016) of spatial changes in the mangrove extents in Sierra Leone coastal landscape complex (SLCLC). Since one of the primary objectives of this study is to inform WA BiCC project for effective and sustainable coastal management, we use variable buffers (1 km, 2.5 km, and 5 km from the coastline) to identify potential ‘deforestation hotspots’ that might require immediate attention from policy-makers. The other objective is to develop a landscape monitoring method using freely available data that can be easily deployed for other WA BiCC countries. Hence we take advantage of the freely available Landsat images and recent advancement in the cloud computational techniques. We utilize Landsat-5, 7, 8 and Sentinel-2 images along with field data to quantify and interpret changes in mangrove covers in the SLCLC. As a requirement of the WA BiCC project, we specifically focus on four estuaries (Scarcies River Estuary, Sierra Leone River Estuary, Yawri Bay and Sherbro River Estuary) for addressing the following questions:

1. How has the mangrove extent changed in the SLCLC over the past 26 years? Is there net mangrove gain or loss?
2. Where did mangrove forests undergo the most changes—closer to the coastline or further away?
3. Are there spatial differences in mangrove changes, e.g., northern SLCLC vs. southern SLCLC?

## 2. Materials and Methods 

### 2.1. Study Area

**Figure 1:**
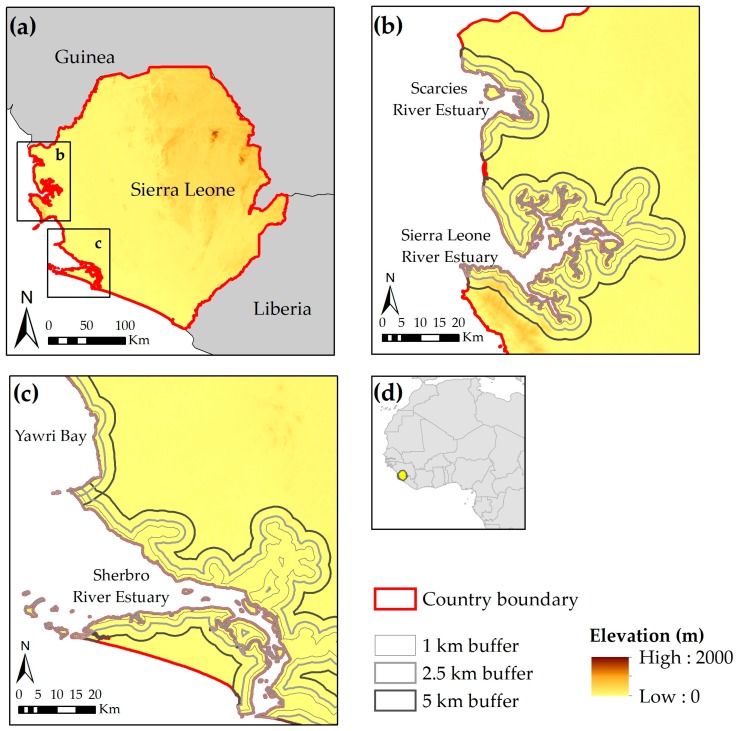
Location of the study area showing: (**a**) Regional topography with focus areas in northern Sierra Leone and Southern Sierra Leone defined by rectangles; (**b**) Focus areas in northern Sierra Leone (Scarcies River Estuary and Sierra Leone River Estuary) with multiple buffers extending inland from the estuary boundary; (**c**) Focus areas in southern Sierra Leone (Yawri Bay and Sherbro River Estuary) with multiple buffers extending inland from the estuary boundary; (**d**) Location of Sierra Leone in West Africa.

Sierra Leone covers 72,300 km^2^ land area and hosts approximately 6.5 million people [46]. This West African country has a tropical climate with two pronounced seasons: a wet season from May to October, and a dry season from November to April. The average temperature also demonstrates a well-defined seasonal cycle, with a maximum around March, and a secondary maximum around October/November, separated by lower temperatures during the rainy season. The specific orientation of the coast in Sierra Leone—perpendicular to the moisture-bearing winds—combined with the regional topography (Figure 1) makes the coast spanning Guinea, Sierra Leone and Liberia the wettest part of West Africa, and among the wettest regions in the world, with rainfall exceeding 4000 mm/year. A relatively long rainy season with high rainfall amounts and the topography result in seasonal influxes of freshwater via twelve major river basins, all draining in the Atlantic Ocean.

Sierra Leone has diverse ecosystems including lowland rainforest, mountain forest, mangrove/coastal ecosystems, freshwater swamps, and marine ecosystems [47]. Once a much forested (70%) country, Sierra Leone now hosts only 5% of the original intact forest [48]. While 75% of the land is arable [47], the most fertile among these ecosystems is the coastal plains including mangrove swamps, riverine grasslands, inland valley swamps, and flood plains of the major rivers. Seasonal wetlands that span from the uplands in the north to the mangroves along the coastline are important habitats for migratory water fowl, water dependent amphibian and mammal species, and serve as grazing lands for waterbuck and buffalo. 

The Sierra Leone coastline stretches for about 506 km and the continental shelf extends for about 27,500 km^2^. This considerable continental shelf, combined with the local currents, creates a substantial upwelling that places Sierra Leone within one of the world’s most productive marine ecosystems [49,50]. Fisheries represent the major source of income (~10% of the GDP) and contribute significantly to poverty reduction and food security in Sierra Leone. These fisheries also support a secondary economy of boat building, wood cutting, transporting fish, weaving baskets, selling fishing gear and petty trading. Around 40,000 artisanal fishers and their families operate about 12,000 fishing boats leading to employment of 500,000 people in the fisheries sector. Most of the artisanal fishing activities occur around the estuaries of three rivers, the Scarcies, Sierra Leone and Sherbro, as well as around Yawri Bay [51], that are also largely covered with mangroves. The five most dominant mangrove species in the region are *Avicennia germinans*, *Rhizophora racemosa*, *Rhizophora harrisonii*, *Laguncularia racemosa*, and *Rhizophora mangle* [52]. 

In this study we specifically focus on multiple buffers extending inland from four estuaries—Scarcies river estuary, Sierra Leone river estuary (SLRE), Yawri Bay, and Sherbro river estuary (Figure 1) because of their varying ecology, mangrove usage, and WA BiCC focus. In Sierra Leone people are heavily dependent on fuelwood for domestic energy, mostly for cooking. Mangrove wood is additionally used for fish processing, building construction poles, and household furniture. While both Scarcies and SLRE have all five dominant mangrove species, Yawri Bay and Sherbro have only three of them. The *Rhizophora* species has been heavily harvested for fuelwood for fish smoking, whereas the *Avicennia* species is primarily harvested for fuelwood for salt processing and experiences less exploitation [51]. 

### 2.2. Satellite Data 

**Figure 2:**
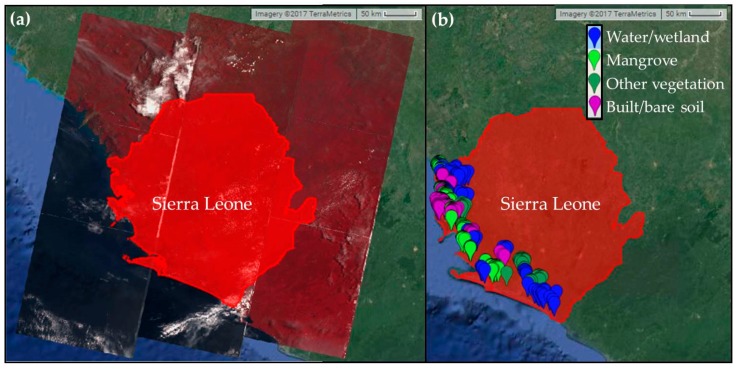
Maps from Google Earth Engine (GEE) showing: (**a**) Landsat footprints covering Sierra Leone; and (**b**) location of validation points used for accuracy assessment for the 2016 classified map.

In order to quantify the spatial extent of the mangroves in selected areas and its temporal changes over several decades a suite of Landsat images were analyzed and compared for the years 1990, 2000, 2010 and 2016 (Table 2). A total of nine Landsat scenes are required to cover the entire country (Figure 2), although more scenes were considered for each year in order to minimize missing data due to frequent cloud cover (Table 2). For the most recent year (i.e., 2016), Landsat 8 OLI images were used on Google Earth Engine (GEE), a cloud computing platform. Landsat 5 images were used for 2010 and 1990, whereas Landsat 7 images were used for 2000 (Table 2)—all available on the GEE platform. A closer look at the image availability revealed that only winter/dry seasons have images available across the years. Since the years considered in this study have varied cloud coverage, a longer time-frame (with two dry seasons) was required for 2000 and 1990 to minimize missing data (e.g., see [1]). All remote sensing analyses were performed on the GEE platform, and classified image covering only the study region were downloaded for further analyses.

The images available on GEE provide calibrated, orthorectified, top-of-atmosphere reflectance values for each band (spatial resolution: 30 m for visible and infrared bands). Calibration coefficients are either extracted from the image metadata or derived from [53]. Both Landsat 5 and Landsat 7 images include a quality mask band identifying pixels as clear (0), water (1), shadow (2), snow (3), or cloud (4) [54,55,56]. Pixels with mask values for shadow, snow, or cloud in this cloud mask band were excluded. At the time of this analysis, Landsat 8 images were not available with a cloud mask band on the GEE platform; hence, a cloud mask function was used to identify pixels with less than 25% cloud cover. Finally, an image composite was created for each year using all visible and infrared bands. For further data reduction, the “median” function on GEE was used that calculates the median value of all unmasked pixels in the input imagery for each pixel location in the output image for each band. Hence, each of these median images is devoid of noise as reflected in extremely high or low values for each band. The median image was then clipped for the low elevation coastal zone (LECZ) with elevation <=40 m, a criteria widely used to define the LECZ (e.g., see [45]).

### 2.3. Land Cover Classification

**Figure 3:**
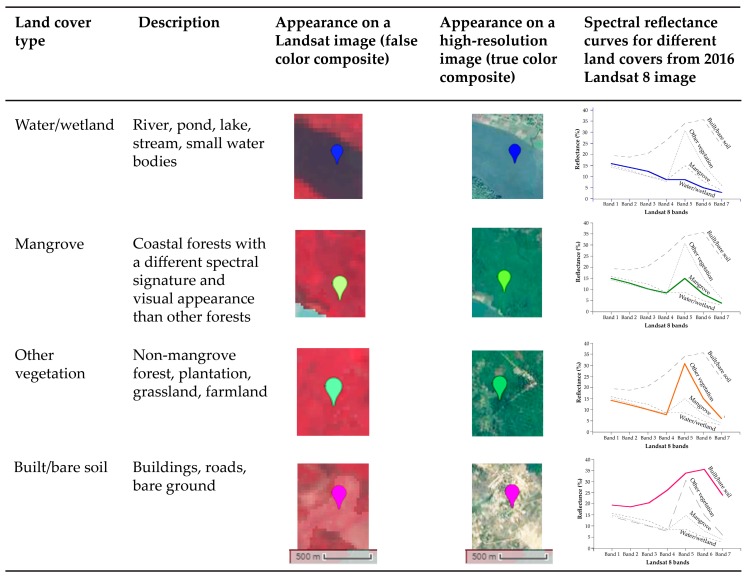
Description of the land cover classes, their appearances on Landsat and high-resolution images, and spectral reflectance curves for representative pixels from each class.

We identified four dominant land covers in the study area using Sentinel-2 and high-resolution images available on the GEE platform—(1) water/wetland, (2) mangrove, (3) other vegetation, and (4) built/bare soil. In the interest of a consistent definition that can be applied across WA BiCC countries, we broadly define ‘mangrove’ as the forest cover type that (i) has a distinct spectral signature compared to other vegetation (Figure 3) and (ii) is located within the LECZ.

We then used the GEE ‘clusterer’ package to classify the clipped composite images for each year. The package offers different clustering algorithms, including k-means, x-means, cobweb, and Learning Vector Quantization, that can classify an image with no *a priori* knowledge. We used a k-means approach [57] to perform an unsupervised classification, as several past studies have successfully utilized unsupervised classification approach for mangrove mapping [14,19,24,25,31,39,45]. On GEE, 50 clusters were first identified in each of the four composite images corresponding to the four time-steps based on the reflectance values from all bands. Then the clusters were sorted based on the mean values for the near-infrared band (band 5 for Landsat 8/band 4 for Landsat 5 and 7), as that band provides the maximum separability across land cover classes (Figure 3). This automated unsupervised classification technique on the cloud computing platform is ideal for similar studies in other developing countries where resources for advanced methods and detailed field data are often unavailable. The sorted clusters were then labeled one of the four land cover classes by visual inspection of the high-resolution images (Figure 3). All analyses were carried out on the GEE platform except class labeling that was done on ArcGIS 10.4.1.

Following the image classification, areas under each land cover were quantified using GIS tools for the four focus areas (Figure 1). We used a GIS shape file for the estuaries and created three buffers from these estuary boundaries that extend 1 km, 2.5 km, and 5 km inland. Buffer zones are not mutually exclusive, i.e., the 5 km buffer encompasses the 1 km and 2.5 km zones. In these buffers we quantified the land cover extents (in absolute numbers and percentages) for each focus area by time slice (1990, 2000, 2010, and 2016) as well as change in land cover between time slices.

## 3. Results

### 3.1. Accuracy Assessment

A total of 300 validation points following a stratified random sampling strategy by area (150 for mangrove and 50 for each of the other three land cover class stratum, randomly distributed across the four focus areas) were collected using Sentinel-2 and other high-resolution images from winter/spring of 2016 on the GEE platform to perform an accuracy assessment for the 2016 classified image. Field data on mangrove locations collected during summer 2016 provided information regarding the appearance of mangroves on high-resolution images, and thus facilitated validation point collection on the GEE platform. The validation points were used to extract land cover classes from the 2016 classified map in ArcGIS. Then a confusion matrix was created with the observed and predicted/classified land cover classes. The overall accuracy (i.e., correct predictions/total predictions) of the 2016 classification is 95% (Table 3). The kappa statistic (κ) for the classified map is 0.93. While we did not directly validate land cover maps from the older dates due to lack of validation data, we used other available estimates to compare the mangrove extents. 

### 3.2. Estimated Mangrove Extents in 2016

**Figure 4:**
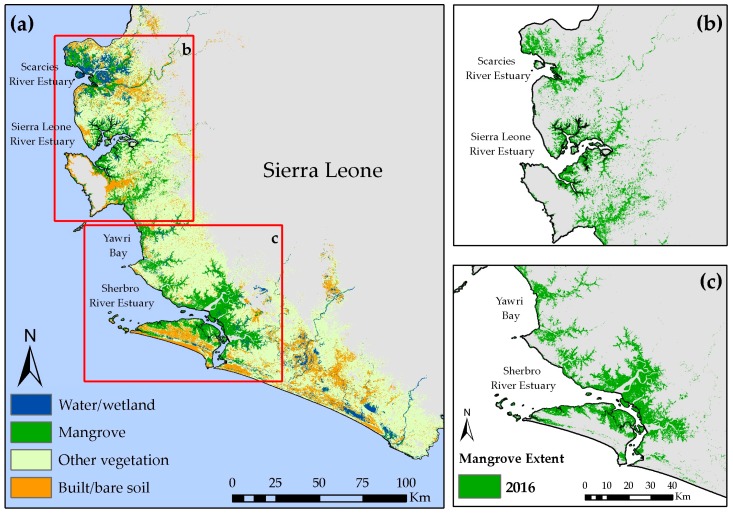
(**a**) Spatial distribution of the four land cover classes across the Sierra Leone coastal landscape complex for 2016. Panels show mangrove extents in (**b**) northern Sierra Leone (Scarcies River Estuary and Sierra Leone River Estuary) and (**c**) southern Sierra Leone (Yawri Bay and Sherbro River Estuary).

Figure 4 shows the spatial distributions of different land covers in the SLCLC. In the SLRE and Sherbro regions the area covered by mangroves increases twofold between 1 km and 5 km buffer, whereas the Scarcies and Yawri bay regions have 5- or 7-times more mangroves when areas further away from the coastline are considered (Table 4). The Sherbro region has the largest area covered with mangroves among the four focus areas, while mangrove cover has the smallest absolute extent in the Scarcies region (except within 1 km buffer). Further investigation of the spatial distribution of the mangroves shows that in SLRE and Sherbro regions considerable portions of the coastline is covered with mangroves (Figure 4b,c) whereas in Yawri Bay mangroves are concentrated along the main rivers (Figure 4c). 

### 3.3. Land Cover Changes within the Focus Areas

**Figure 5:**
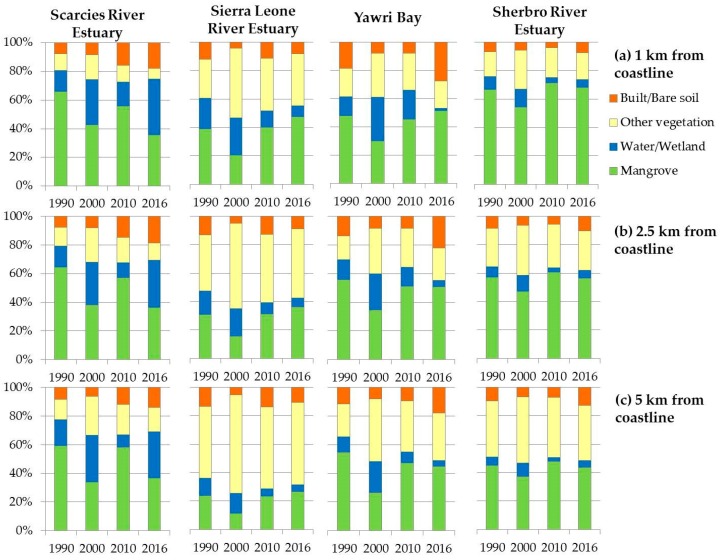
Relative extents of different land covers within the four focus areas during 1990–2016. Panels show mangrove extents within buffers of (**a**) 1 km, (**b**) 2.5 km, (**c**) 5 km extending inland from the coastline.

Figure 5 further summarizes the spatiotemporal changes of the different land cover classes between different time slices. In the Scarcies region, mangroves remained the most dominant land cover (Figure 5). However, the Scarcies witnessed fluctuations in mangrove extents with more mangroves during 1990 (66% within 1 km, 65% within 2.5 km, 59% within 5 km) and 2010 (56% within 1 km, 57% within 2.5 km, 58% within 5 km). Mangrove extents declined in this region during 2000 (43% within 1 km, 38% within 2.5 km, 34% within 5 km) and 2016 (36% both within 1 km and 2.5 km, 37% within 5 km), mostly owing to an increase in water/wetland (Figure 5). Built area/bare soil more than doubled during post-2000 years, increasing from 8% to 18% both within 1 km and 2.5 km, and from 6% to 14% within 5 km.

In the SLRE region, other vegetation (e.g., forest, plantation, farmland, etc.) has been the most dominant land cover over the past 26 years, except for the 1 km buffer zone where mangrove was the most dominant land cover in the years of 1990, 2010 and 2016 (Figure 5). Other vegetation coverage ranged between: (i) 27% in 1990 and 49% in 2000 within the 1 km buffer, (ii) 39% in 1990 and 60% in 2000 within 2.5 km buffer, and (iii) 51% in 1990 and 69% in 2000 within 5 km buffer. Water/wetland coverage ranged between 5% and 21% across years and buffer zones, with more coverage closer to the coastline. Built area or bare soil ranged between 4% and 14% across space and time, with minimum coverage in 2000 and maximum coverage in 2010.

In the Yawri Bay region, mangroves covered the most area across the years and buffer zones, except for in 2000 with: (i) both wetlands/water and other vegetation covering more area than the mangroves within 1 km buffer, and (ii) other vegetation covering more area in 5 km buffer (Figure 5). Water/wetland has declined over time (e.g., 14% in 1990 to 2% in 2016 within 1 km) within all three buffer zones, with an increase during 2000. Built area/bare soil increased over time (19% to 27% within 1 km, 14% to 22% within 2.5 km, and 11% to 18% within 5 km), with a decline in between for all three buffer zones.

As with the Scarcies region, mangroves remained the dominant land cover in the Sherbro region with an estimated area covering 68% within 1 km, 56% within 2.5 km, and 44% within 5 km buffer zones in 2016 (Table 4). The only exception is within 5 km buffer zone in 2000 (Figure 5), when other vegetation covered more area (46%) than mangroves (37%). The other two land covers remained relatively stable throughout the years, and covered less area (~10%) compared to mangroves and other vegetation. The findings from the field visits during summer 2016 showed that the Sherbro area is covered with largest and oldest trees, indicating less degradation over past decades than in other areas and explaining a relative stability of the mangrove cover over time.

### 3.4. Spatiotemporal Changes in Mangrove Extents over the Last Three Decades

Table 5 shows the overall change in mangrove cover between 1990 and 2016 in each focus area for different buffers. The largest decline is observed in the Scarcies for all buffers, up to 46% in the 1 km buffer. The SLRE has experienced an increase of about 22% in the 1 km buffer. The Sherbro and Yawri Bay regions experienced slight expansion (<10%) of the mangroves when the narrowest buffer, closest to the sea shore is considered. These two regions witnessed modest decline (<20%) when larger buffers are considered. This comparison, however, does not capture the full history of fluctuations. 

As shown in Figure 5, mangroves’ relative extent is the lowest in 2000 in each region and each buffer, which is also the year where the other types of vegetation and water/wetland combined cover the largest area. Subsequently, in 2010 the relative mangrove extent increases back to the extents from 1990 in nearly all cases, with the exception of Yawri Bay. Then it remains stable in Sherbro and Yawri Bay, while it continues to increase in SLRE. In the Scarcies, on the other hand, it decreases again in 2016 to the extent similar to that in 2000.

**Figure 6:**
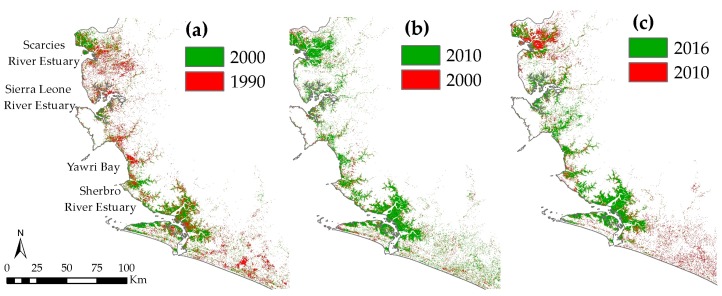
Decadal changes in mangrove extents in the Sierra Leone coastal landscape complex (SLCLC) during (**a**) 1990–2000, (**b**) 2000–2010, (**c**) 2010–2016.

Figure 6 further highlights decadal changes in mangrove extents in the SLCLC. The largest loss of mangroves in the Scarcies region is observed between 1990 and 2000 and is mostly located in the south-eastern part of the Scarcies region and along the Greater Scarcies River (Figure 6a), while the loss between 2010 and 2016 mostly happened in the northern part of the region (Figure 6c). In other parts of the SLCLC, signs of non-clustered deforestation are present in pre- and post-2000 years, with the exception of Southern Yawri Bay. In general, the Scarcies and Yawri Bay seem to undergo largest mangrove fluctuations between years.

## 4. Discussion and Conclusions

This study, based on a discrete classification of Landsat pixels, provides an estimate of 1526.42 km^2^ mangrove extent for 2000 that is similar to the estimates provided by [1]. While a continuous classification approach can, as argued by [41], provide more accurate estimates, it is almost impossible to apply for historical land cover analyses in the absence of existing maps with known accuracy or extensive field data from earlier years. Therefore, it could not be applied in our study aimed at estimating the evolution of the mangrove extents in Sierra Leone. Our study relies solely on dominant spectral signature within a single pixel (30 m × 30 m) for a discrete classification, i.e., presence/absence of a certain class. While it is possible that our study overestimates mangrove coverage in the SLCLC because of the way discrete classification works, our approach allows us to provide important insights into decadal changes in spatial patterns in mangrove forests. Besides, our findings agree with those from the past studies that showed an increase in mangrove extents after 2000 [5,41]. 

As shown in Figure 5 and Figure 6b, all four focus areas witnessed an increase in mangrove extents in all three buffer zones between 2000 and 2010. However, only SLRE witnessed an increase in mangrove area post-2010, most probably as a result of reforestation efforts. Considering the overall changes during 1990–2016, mangroves have declined only in the farthest buffer in the Sherbro and Yawri Bay regions, pointing to the possible reclaiming of the land and expansion of agriculture by the communities living inland. The Scarcies region underwent the most extensive mangrove loss among all the areas (Table 5), but in different locations for pre-2000 and post-2010 time periods (Figure 6). In both cases the most probable cause is the conversion of mangroves to rice paddies. However, the rapid recovery of the mangroves over a period of 10 years between 2000 and 2010 in some areas is more indicative of degradation or ‘thinning out’ during the periods prior to 2000 rather than a complete deforestation. While causality is difficult to attribute, the decade of the 1990s was also a period of civil war, which may have heightened dependence on mangrove forests for fuel wood, charcoal production, and construction, and/or weakened conservation and protection measures. These findings, especially the overall patterns in change, can particularly benefit regional and/or national policy-makers in drafting coastal conservation policies.

It is important to note that it is challenging to distinguish between wetlands and sparse mangrove forests, because of the inherent limitations of optical remote sensing in terms of the spectral and spatial resolution, especially in tropical countries with frequent cloud cover. Hence, it is possible that a pixel with sparse mangrove cover will record a spectral signature of the underlying water that is dominant within that pixel and thus be classified as water/wetland. A validation check with high-resolution images from 2016 indeed confirms that the area along and surrounding the Scarcies river hosts young and sparsely distributed mangroves, rather than a complete clear-cut of dense mangrove forests. Even though high-resolution satellite images do not exist for earlier years for validation purposes, it is possible that mangrove forests in the Scarcies region witnessed degradation or ‘thinning out’ during 1990–2000 rather than a complete deforestation. Thus, the fluctuations in mangrove extents as shown in Figure 6 possibly represent alternate thinning and reforestation. This is consistent with the high regeneration potential observed on field visits to the Scarcies region.

To be useful at large scale, satellite-based assessment of mangrove cover needs to be complemented by in-situ evaluation of the quality of the forests. The field trips during summer 2016 indicate that, despite degradation, the remaining mangroves in the Scarcies region show higher species diversity (relative to other regions in Sierra Leone), and high regeneration level; thus indicating human pressure on the forests, but also high regeneration potential should human pressures be lowered or better managed. This is further supported by the fluctuations in mangrove forest cover, from dense to sparse to dense, estimated in this study. The Sherbro area is on the opposite side of the spectrum, with lowest species diversity, highly dominated by *Rhizophora racemosa*. These mangroves are also the oldest among the four areas and have the lowest regeneration rates with little disturbance in the forests. These forests, while with high commercial potential, exhibit low adaptation potential to future, potentially altered climatic conditions. The SLRE has the smallest trees with the lowest basal area pointing to youngest forests, sign of past and current exploitation of the forest as well as recent reforestation efforts. The Yawri Bay has fewer adult trees but the highest number of seedlings, both showing signs of good potential for regeneration and sustainability. 

Overall, despite a noticeable overall long term decrease of the mangrove cover in Sierra Leone a closer look at mangrove evolution shows a good potential for conservation if properly monitored and managed. Conserving the fluctuating mangroves in the SLCLC would require a carefully designed management plan involving current and alternative livelihood strategies, sustainable resource management schemes at local and national levels and sensitization of the populations about mangrove services and their value under climate change, beyond responding to communities’ immediate needs. Among the coastal populations, fishing communities are the ones that will be the most affected by the decline in mangroves, yet are also the ones who currently rely on mangroves the most for their livelihoods. These populations exhibit low livelihood diversification mostly revolving around fishing and fish processing with some contribution from agriculture and petty trade [52]. The SLRE has slightly higher diversification of livelihood strategies, largely due to the proximity of Freetown and slightly higher grounds. In the Scarcies and Sherbro regions alternative or supplementary livelihood opportunities are quasi non-existent. In most of the communities visited during field trips, mangroves are also open-access and individuals can access ownership by ‘adding value’ to the land, which in most cases translates into clearing the mangroves for rice cultivation. Existing community-based management initiatives seem to be mostly geared towards short term economic benefits from lands covered by mangroves, rather than driven by holistic, long term plans that include a wide range of services provided to a wide range of populations that would insure sustainability of the resource and fishing livelihoods. While projects aimed at mangrove conservation could benefit from the existing management structures, significant change to the management goals would be required.

## Figures and Tables

**Table 1:** Comparison of mangrove extents in Sierra Leone for 2000 as estimated by the Mangrove Forests of the World (MFW), and the continuous mangrove forest cover for the 21st century (CGMFC-21).

Dataset	Source	Brief Description	Area (km^2^)
MFW	Giri et al., 2011 [1]	Landsat-derived discrete classification	1564.96
-	Fatoyinbo and Simard, 2013, [45]	Landsat-derived discrete classification	955
-	FAO, 2007 [5]	Country-specific reports based on their own classification system	1053
CGMFC-21—Revised MFW	Hamilton and Casey, 2016 [41]	Integration of discrete MFW dataset and continuous Global Forest Cover (GFC) dataset	655.67
CGMFC-21—Revised Terrestrial Ecoregions of the World (TEOW)	Hamilton and Casey, 2016 [41]	Integration of discrete TEOW dataset and continuous Global Forest Cover (GFC) dataset	2917.01

**Table 2:** Details of the satellite images used in the study.

Sensor	Study Period	Image Dates	No. of Total Images
Landsat 8	2016	December 2015–May 2016	99
Landsat 5	2010	November 2009–May 2010	46
Landsat 7	2000	November 1999–December 2000	120
Landsat 5	1990	November 1989–December 1990	34

**Table 3:** Accuracy assessment for the 2016 classified map. The overall accuracy is shown in bold.

	Ground Truth				
Classified	Water/Wetland	Mangrove	Other Vegetation	Bare/Built	User’s Accuracy
Water/wetland	46	1	0	0	98%
Mangrove	4	143	0	3	95%
Other vegetation	0	6	50	1	88%
Bare/built	0	0	0	46	100%
Producer’s accuracy	92%	95%	100%	92%	95%

**Table 4:** Estimated mangrove cover in 2016 within different buffers for the four focus areas in Sierra Leone.

Region	1 km Buffer		2.5 km Buffer		5 km Buffer	
	Mangrove Area (sq. km)	Relative Mangrove Extent	Mangrove Area (sq. km)	Relative Mangrove Extent	Mangrove Area (sq. km)	Relative Mangrove Extent
Scarcies River Estuary	17.35	36%	42.38	36%	90.39	37%
Sierra Leone River Estuary	160.71	48%	249.01	36%	290.55	26%
Yawri Bay	15.43	51%	55.05	51%	113.98	45%
Sherbro River Estuary	355.78	68%	605.54	56%	762.99	44%

**Table 5:** Summary of mangrove extents (area in sq. km.) during 1990 and 2016 for the four focus areas in the Sierra Leone coastal landscape complex. Values in bold denote mangrove gain, while values in red italic denote mangrove loss during 1990–2016. The ‘Sierra Leone coastal landscape complex’ refers to all the mangroves along the full length of the coast, not only in the buffers in the four areas.

Region	1 km Buffer			2.5 km Buffer			5 km Buffer		
	1990	2016	Change (Relative Change)	1990	2016	Change (Relative Change)	1990	2016	Change (Relative Change)
Scarcies River Estuary	31.95	17.35	*−14.60 (−46%)*	73.43	42.38	*−31.05 (−42%)*	142.58	90.39	*−52.19 (−37%)*
Sierra Leone River Estuary	131.87	160.71	**28.84 (22%)**	213.02	249.01	**35.99 (17%)**	261.69	290.55	**28.86 (11%)**
Yawri Bay	14.34	15.43	**1.09 (8%)**	60.11	55.05	*−5.06 (−8%)*	138.30	113.98	*−24.32 (−18%)*
Sherbro River Estuary	336.82	355.78	**18.96 (6%)**	591.48	605.54	**14.06 (2%)**	768.26	762.99	*−5.27 (−1%)*
	1990	2016	Overall change (Relative change)						
Sierra Leone coastal landscape complex	2434.82	1834.32	*−600.5 (−25%)*						
